# Ketamine and rapid antidepressant action: new treatments and novel synaptic signaling mechanisms

**DOI:** 10.1038/s41386-023-01629-w

**Published:** 2023-07-24

**Authors:** John H. Krystal, Ege T. Kavalali, Lisa M. Monteggia

**Affiliations:** 1 Department of Psychiatry, Yale School of Medicine, New Haven, CT USA; 2 Department of Pharmacology and the Vanderbilt Brain Institute, Vanderbilt University, Nashville, TN USA

**Keywords:** Depression, Depression

## Abstract

Ketamine is an open channel blocker of ionotropic glutamatergic *N*-Methyl-*D*-Aspartate (NMDA) receptors. The discovery of its rapid antidepressant effects in patients with depression and treatment-resistant depression fostered novel effective treatments for mood disorders. This discovery not only provided new insight into the neurobiology of mood disorders but also uncovered fundamental synaptic plasticity mechanisms that underlie its treatment. In this review, we discuss key clinical aspects of ketamine’s effect as a rapidly acting antidepressant, synaptic and circuit mechanisms underlying its action, as well as how these novel perspectives in clinical practice and synapse biology form a road map for future studies aimed at more effective treatments for neuropsychiatric disorders.

## Introduction

Psychiatric disorders and neurotherapeutics are exhibiting a renaissance of new treatments for mood disorders that engage novel synaptic signaling mechanisms. The fertility of this moment is illustrated by the U.S. Food and Drug Administration (FDA) approvals of Esketamine (Spravato) and Brexanolone (Zulresso) in 2019. Further, the FDA approval of Esketamine has seemed to open the door to impending FDA approvals of other consciousness-altering medications including psilocybin and methylenedioxymethamphatemine (MDMA). This impressive concentration of emerging novel treatments has been unmatched since 1957, the year that the last mechanistically novel antidepressants were discovered, the monoamine transporter antagonists and the monoamine oxidase inhibitors [1–4]. The need for these new antidepressants is unquestionable. Despite over 60 years of antidepressant development, mood disorders remain among the most disabling medical conditions in the world and they are associated with shortening of life expectancy by up to 10 years [5, 6]. Inadequate antidepressant efficacy is clearly a contributor to the burden posed by mood disorders as the NIMH STAR*D study indicated that only approximately one-third of depressed patients remitted on their initial antidepressant, modest incremental benefits were seen with subsequent treatments, approximately one-third of patients remained unremitted after four treatments, and the one-year relapse rate among patients with treatment-resistant symptoms (Steps 3 and 4) was approximately 75% [7, 8]. While opportunistic observations contributed to the discoveries of the antidepressants in both the late 1950’s and the modern era, the scientific context of the discovery of the antidepressant efficacy of ketamine was fundamentally different from the earlier discoveries [9]. The following review will consider the neuroscience framework that led to the discovery of the antidepressant effects of ketamine and the resulting explosion in translational neuroscience devoted to understanding the growing array of synaptic signaling mechanisms through which its antidepressant effects are expressed. We will also synthesize several of the seemingly disparate findings regarding ketamine’s mechanism of action including the NMDA receptor dependence on excitatory neurons compared to inhibitory neurons, the glutamate surge that has been reported following NMDAR block, the intracellular signaling pathway, the requirement for BDNF-TrkB activity, and the role for postsynaptic AMPA receptors. Other proposed mechanisms for ketamine’s antidepressant action include ketamine enantiomers [10] and metabolites [11], the potential role of inflammatory processes [12], and non-neuronal contributions including those from microglia [13], among others.

## From the monoamine hypothesis of depression to NMDAR antagonist treatments

In the 1970s through the 1990s, Yale University was a hotbed of preclinical and translational research attempting to characterize disturbances in monoamine signaling contributing to the neurobiology of depression and the mechanisms through which monoamine signaling-targeting antidepressants produced their therapeutic effects. Studies led by Dennis Charney, Pedro Delgado, George Heninger, and others clearly implicated ongoing monoaminergic availability in monoaminergic antidepressant efficacy. In depressed patients who had responded to antidepressant treatment, depletion of either serotonin or norepinephrine prevented or transiently reversed the antidepressant effects of serotonin transporter antagonists or norepinephrine transporter antagonists, respectively [14–16]. However, subsequent Yale and NIMH studies attempting to produce depression in healthy people by depleting serotonin, norepinephrine, or both were not successful [17–21]. While other findings in the field suggested that monoamine signaling was relevant to the biology of depression, the mixed findings from the depletion studies suggested that the framework for the biology of depression needed to be broadened to encompass other signaling mechanisms [22].

In parallel, one of us (J.K.) developed R,S-ketamine infusion as a psychopharmacology research platform to probe NMDAR function relevant to cognitive function, psychosis, and dissociation in healthy humans [23]. This work was motivated by parallel broadening of perspective from the “dopamine model” of schizophrenia to one involving the intrinsic signaling mechanisms of the cortex, i.e., glutamate and GABA [24]. It identified an optimal ketamine dose to elicit behavioral changes without producing delirium or anesthesia, i.e. a 40-minute intravenous infusion of 0.5 mg/kg, targeting plasma ketamine levels of 130–200 ng/ml. At comparable doses of S-ketamine, its effects on negative symptoms correlate with in vivo occupancy of NMDARs [25].

The convergence of the broadening perspective of the biology of depression with the development of an approach to probe glutamate synaptic function resulted in the discovery of the rapid antidepressant effects of ketamine. The initial pilot study used the previously ketamine infusion strategy to conduct the first randomized, placebo-controlled test of ketamine response in depression [26]. This study built on inconclusive findings with lower potency NMDAR antagonists including amantadine and memantine [27]. The investigative team also was aware of earlier studies in animals that described antidepressant effects of NMDAR partial agonists and antagonists [28, 29]. However, none of these studies predicted the distinctively rapid and robust antidepressant effects of a single ketamine dose in depressed patients observed in the initial pilot study, nor the efficacy in treatment-resistant symptoms of depression, bipolar disorder [30] or suicide ideation [31].

Subsequent research validated the safety and efficacy of R,S-ketamine and Esketamine for the treatment of a growing array of psychiatric disorders. When used to treat depression, ketamine is dosed with the minimal frequency possible. Although clinical practices may vary [32], it is generally started with twice weekly dosing for the first month, weekly for the following month, and then with reduced frequency thereafter among patients engaged in maintenance treatment. R,S-ketamine has shown efficacy for depression in the context of treatment-resistant depression [30], bipolar disorder [33], OCD [34], and PTSD [35]. R,S-ketamine appears to have long-term safety and efficacy [36, 37]. Beyond depression, R,S-ketamine produces clinical response in patients with anxiety disorders [38] and improvement in social and vocational function in patients diagnosed with borderline personality disorders [39]. Esketamine studies have documented the long-term safety and efficacy of Esketamine using randomized placebo-controlled [40–42] and drug-discontinuation [43] designs. Importantly, the drug discontinuation study indicated that among responders to the combination of a new antidepressant and Esketamine, maintenance of Esketamine with the new antidepressant halved the relapse rate relative to maintenance the new antidepressant by itself. In other words, Esketamine appeared to produce a larger protective effect against relapse over one year relative to antidepressant treatment than antidepressants typically show when compared to placebo [44]. To date, there have not been well-powered head-to-head comparisons of R,S-ketamine and Esketamine. Meta-analysis appears to give a small edge to intravenous R,S-ketamine [45], but cross-trial comparisons must be interpreted very cautiously due to unaddressed confounds. An observational study did not find differences in efficacy between intravenous R,S-ketamine and intransal Esketamine, but did suggest that R,S-ketamine acted more rapidly [46]. Thus, conclusions regarding the relative efficacy of these treatments are premature.

NMDAR antagonists studied to date appear to have a narrow dose window for optimized clinical response. Three randomized placebo-controlled studies in treatment-resistant depression and PTSD [35, 47–49] suggest that lowering the dose by approximately half or more (≤0.2 mg/kg) is associated with loss of antidepressant efficacy, while doubling the ketamine dose (1.0 mg/kg) increases the intensity of dissociative symptoms without augmenting efficacy. At anesthetic doses (doses ≥2.0 mg/kg), ketamine does not appear to produce antidepressant effects [50]. The sensitivity of response rates to small dose differences was also evident for Esketamine, where a study in geriatric patients was ineffective overall, an outcome that might have been related to starting older patients on a low ineffective dose of 28 mg. instead of the usual 56 mg dose [51]. Because of the high degree of sensitivity to ketamine dose, NMDAR antagonist treatment strategies that do not appear to produce comparable leves of NMDAR antagonism as the standard ketamine infusion paradigm [52] may not engage the same forms of neuroplasticity as the antidepressant doses of R,S-ketamine and Esketamine.

There may be specific roles for psychotherapies combined with ketamine or Esketamine treatment. Ketamine, due to its primary action as a NMDAR blocker, is known to interfere with NMDAR-dependent forms of Hebbian neuroplasticity [53, 54]. This has led to studies suggesting a capacity of ketamine to disrupt the reconsolidation of maladaptive memories contributing to PTSD [55] and addiction [56]. In other words, by interfering with memory reconsolidation, ketamine may reduce the negative impact of trauma memories in PTSD or drug-related cravings in addiction. These studies build on preclinical research suggesting that if ketamine is administered in association with the reexposure to prior threat [57, 58] or rewarding drugs [59], the reconsolidation of fear or drug-related memories was attenuated. Rather unexpectedly, ketamine has also been shown in rodent models to elicit homeostatic neuroplasticity within an hour following administration [60], which has been linked to its rapid antidepressant action. This raises an intriguing question of whether ketamine is producing beneficial effects by different neuroplasticity processes in patients with depression compared to PTSD or substance use disorders. Pilot studies have described beneficial effects where ketamine has been combined with CBT [61] and a digital intervention. The combination of ketamine with psychotherapies is an active area of investigation and may broaden the population of patients who benefit from ketamine treatment.

## Glutamate synaptic dysfunction in major depression (MDD) and PTSD

Glutamate synaptic function appears to be downregulated in cortical circuits in major depression. Glutamate synaptic dysfunction has long been thought to be a contributor to the neurobiology of depression [62]. Proton magnetic resonance spectroscopy (^1^H-MRS) provided the first non-invasive measurements of brain glutamate and glutamine levels, generally reporting reductions in frontal cortex measurements [63], although there are differing findings [64]. These measurements are difficult to interpret mechanistically as these metabolites are present in all cell types within the virtual box of brain tissue (voxel) in which measurements are made. Further, the pool of glutamate available for neurotransmission is a small fraction, perhaps 10%, of neuronal glutamate [65, 66]. These limitations are somewhat addressed using ^13^C-MRS in combination with an isotopically labeled metabolic substrates (^13^C-glucose, ^13^C-acetate), which enable dynamic tracking of the levels of isotopically-labeled metabolites of these substrates and the characterization of the rates cellular processes, such as anaerobic metabolism, aerobic metabolism (tricarboxylic acid cycle activity, V_TCA_), and the rate of conversion of glutamate to glutamine, which is stoichiometrically related to the rate of glutamate release (V_Cycle_) [67–69]. Using these techniques, it was first shown that frontal cortical V_TCA_ but not V_Cycle_ was reduced in medication-free depressed patients compared to healthy subjects [70]. A second study applying this approach to PTSD patients with and without depression reported similar findings, but analyzed the data in a novel fashion that indicated per molecular of glutamate released, the associated metabolic activity was reduced (“energy per cycle”; [71]). Functional studies using event-related potentials assessed with electroencephalography (EEG) and magneto-encephalography (MEG) are consistent with the presence of synaptic downregulation in depression and PTSD [72, 73]. Similarly, functional magnetic resonance imaging describe reductions in cortical functional connectivity associated with these disorders [74].

A second form of glutamate synaptic dysfunction in MDD and PTSD is synaptic loss. In animals, chronic stress exposure produces clear evidence of synaptic pruning [75–77]. Structural MRI studies of MDD had long described volumetric loss from which many inferred the existence of synaptic deficits [78–80]. Post-mortem studies of human post-mortem cortical and limbic tissue also describe altered expression levels for genes coding for pre- and postsynaptic proteins [81, 82]. The advent of a radioligand for the synaptic vesicle protein-2A (SV2A) enabled the first in vivo descriptions of reductions in synaptic density in MDD patients with and without PTSD comorbidity who expressed moderate-to-severe symptoms [83]. In contrast to the presynaptic protein SV2A, imaging of postsynaptic glutamate synaptic proteins is less well advanced. Metabotropic glutamate receptor 5 (mGluR5) is a post-synaptic protein and there are variable findings across studies [84, 85]. A recent study [86] reported no change overall in MDD, but reductions in mGluR5 density associated with depression severity, consistent with synaptic losses in patients with moderate-to-severe depression. Based on the accumulating evidence for deficits in glutamatergic synaptic function as well as structural plasticity of synapses, it is plausible to expect that ketamine’s rapid antidepressant action may either counter or mask these pathological defects.

## Ketamine effects on brain circuits in MDD patients

Since the landmark papers on the mechanisms of action of ketamine [60, 87], there has been a concerted effort to assess the implications of the preclinical findings for the antidepressant effects of ketamine in patients. These two papers put forth different hypothesis on the mechanism of the rapid antidepressant action of ketamine, which we discuss as well as provide a perspective on their commonalities.

**Fig. 1:**
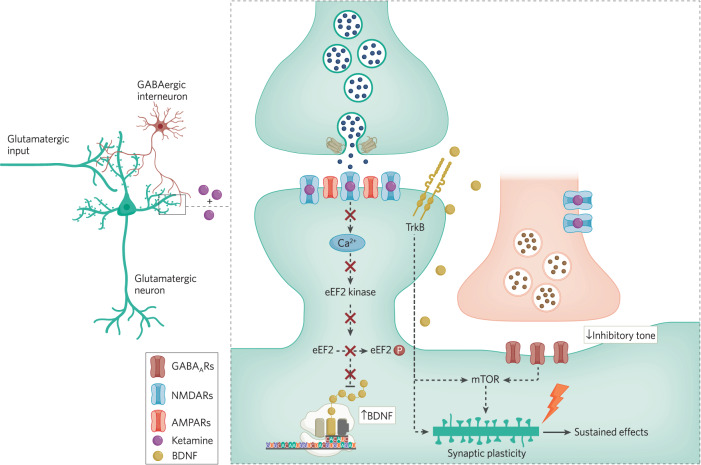
Converging synaptic signaling pathways underlying ketamine action. The figure depicts a basic synaptic circuit where an excitatory pyramidal neuron receives inputs from other excitatory neurons as well as inhibitory neurons. At excitatory glutamatergic synapses onto excitatory neurons, glutamate release and NMDA receptor activation leads to activation of eEF2 kinase, triggering eEF2 phosphorylation and silencing of brain-derived neurotrophic factor (BDNF) translation. Ketamine-mediated NMDA receptor blockade, in turn, ceases tonic eEF2 kinase activity, resulting in a gradual loss of eEF2 phosphorylation and de-suppression of BDNF translation, ultimately triggering TrkB receptor signaling. TrkB signaling and subsequent rapid homeostatic synaptic plasticity is required to elicit not only rapid effects of ketamine but also its sustained effects. In the meantime, ketamine-mediated transient reduction of excitatory drive onto inhibitory interneuron resident NMDARs is postulated to suppress tonic release of GABA and disinhibit activity of the target excitatory neurons. The resulting increase in glutamatergic activity produces downstream activation of mammalian target of rapamycin (mTOR) function to elicit structural plasticity and produce the rapid and sustained antidepressant effects. While the two pathways may act synergistically, it is important to note that mTOR may also acts as a downstream target for TrkB signaling, which may be a point of convergence of the two pathways. These initial synaptic plasticity events trigger sustained effects of ketamine via transcriptional processes. This figure was made by BioRender.

**Fig. 2:**
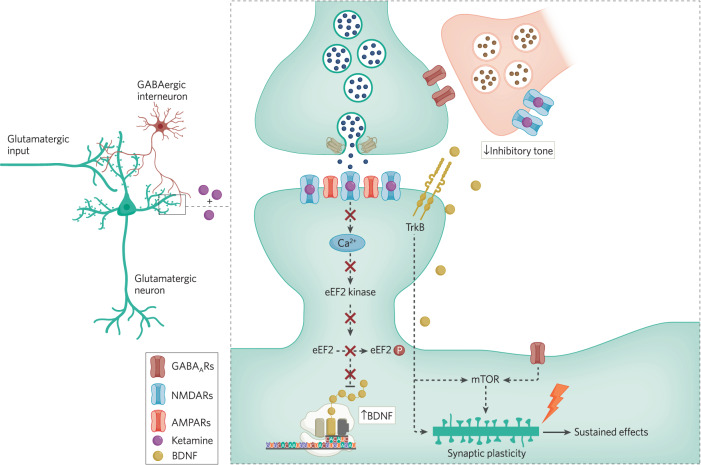
Role of presynaptic disinhibition of glutamate release in ketamine action. The figure depicts an alternative basic synaptic circuit where an excitatory pyramidal neuron receives inputs from other excitatory neurons, where inhibitory inputs regulate the extent of glutamate release from primary excitatory neurons. While this circuit is not mutually exclusive with the one presented in Fig. 1, it highlights the role of NMDA receptors on inhibitory neurons and their impact on regulation of glutamate release. According to this “disinhibition” model, ketamine predominantly acts on inhibitory interneurons, curtailing their tonic NMDAR dependent activity and in turn augmenting excitability of glutamatergic neurons as well as ensuing glutamate release. This increased glutamate release activates postsynaptic AMPA receptors leading to activation of mTOR signaling. This figure was made by BioRender.

Research in the Duman laboratory proposed that ketamine action involves the blockade of NMDARs on inhibitory interneurons [88, 89]. This transient reduction of excitatory drive to these inhibitory interneuron resident NMDARs is postulated to suppress tonic release of GABA and disinhibit activity of the target excitatory neurons. The resulting increase in glutamatergic activity produces downstream activation of mammalian target of rapamycin (mTOR) function to increase dendritic spine formation and produce the rapid and sustained antidepressant effects [87, 90] (see Fig. 1 and Fig. 2). However, it is important to note that disinhibitory effects similar to ketamine action can also be elicited by the closely related NMDA receptor blocker memantine [91], although clinically memantine administration does not induce rapid antidepressant responses [92]. It should be noted, however, that a 40 mg dose of memantine [93], twice the dose of memantine used in the depression trial, still produces subjective effects (euphoria, stimulation, sedation, etc.) that are similar but quite less intense than a standard antidepressant dose of ketamine [94]. Thus, it is not clear whether sufficient memantine doses have been administered to depressed patients to enable an adequate evaluation of its antidepressant efficacy. However, increasing the dose of memantine may result in off-target effects that make it difficult to discern whether it is NMDAR mediated.

The Monteggia and Kavalali labs were also investigating the mechanism of ketamine action and identified a key role for synaptic plasticity, specifically a novel form of rapid homeostatic plasticity, in the behavioral effects. Ketamine produces rapid antidepressant effects that in some patients persist for days to more than a week in some patients. Following administration, ketamine has a short half-life suggesting that its antidepressant effects are due to a form of plasticity that outlast the systemic presence of ketamine. Although ketamine is a well characterized NMDA receptor antagonist, rather unexpectedly it was shown to induce synaptic potentiation in the hippocampus. This potentiation involves block of NMDA receptors and the engagement of intracellular signaling to increase neurotransmission carried out by α-amino-3-hydroxy-5-methylisoxazole-4-propionic acid (AMPA) type glutamate receptors that persists for several hours [60, 95].

While NMDA receptor activation by activity dependent release of glutamate has been traditionally associated with Hebbian forms of plasticity such as Long-Term Potentiation and Long-Term Depression, blockade of NMDA receptors has been shown to elicit a form of homeostatic synaptic plasticity [96]. Unlike Hebbian forms of plasticity, homeostatic plasticities act in a negative feedback manner to counter substantive increases or decreases in activity by re-balancing synaptic strengths. These plasticity forms involve up- or down-regulation of synaptic weights on a given neuron by a multiplicative factor in a process commonly referred to as “synaptic scaling”. Synaptic re-balancing triggered by homeostatic synaptic scaling maintains relative weights of synapses and does not alter the “information” content of synaptic inputs. This feature of homeostatic synaptic scaling makes it an ideal form of plasticity to drive ketamine action as the cognitive functions — that are thought to rely on relative values of synaptic weights to each other — are expected to be unperturbed. Moreover, extensive studies have also demonstrated that homeostatic synaptic plasticity elicited by NMDA receptor block by ketamine occurs rapidly within hours consistent with the time course of ketamine action [60, 95, 97]. To date reverse genetic studies, taking advantage of mice carrying mutations in several signaling nodes along this signaling pathway, suggest that ketamine’s direct effect at synaptic NMDARs is necessary and sufficient to account for the behavioral effects [60, 95, 98].

## Ketamine effects on intracellular signaling to mediate antidepressant action

Earlier studies on the mechanisms of action of classical antidepressant in preclinical models established a strong causal link of BDNF and subsequent signaling via TrkB receptors as a key mediator of antidepressant action [99, 100]. Ketamine’s fast-acting antidepressant action is also dependent on BDNF, specifically rapid translation and synthesis of BDNF protein in hippocampal dendrites [60]. Mice with the BDNF Val66Met polymorphism, which impairs trafficking of BDNF mRNA to dendrites, also show attenuated responses to ketamine [101], although ketamine appears to retain efficacy in humans who are homozygous for the Met allele of *BDNF* [49]. The form of synaptic scaling elicited after ketamine administration discussed above has also been shown to be strictly dependent on BDNF-TrkB signaling [60, 95, 98].

The identification of a key requirement for BDNF-TrkB signaling in ketamine action provided a starting point to investigate the intracellular signaling that produces rapid antidepressant action. This effort lead to elucidation of the essential role of eukaryotic elongation factor 2 and its phosphorylation by its dedicated kinase, eukaryotic elongation factor 2 kinase (eEF2K), in this process.

eEF2 kinase is a calcium-calmodulin-dependent protein kinase (also called CaMKIII), which activity is maintained by a high affinity interaction with calcium-calmodulin complexes. Due to the high affinity nature of this interaction, even resting levels of calcium signaling triggered by spontaneous glutamate release mediated NMDA receptor activation is sufficient to maintain its activity [102]. Detailed analysis of synaptic signal transduction events associated with ketamine action on NMDA receptors provided evidence that ketamine blockade of tonically activated NMDA receptors results in suppression of these resting calcium signals and inhibition of eEF2K activity, resulting in dephosphorylation of eEF2 and de-suppression of dendritic protein synthesis in particular of BDNF. BDNF, in turn, via activation of postsynaptic TrkB receptors promotes the insertion of AMPA receptors, that produces the novel form of synaptic potentiation in the hippocampus that underlies the rapid antidepressant action [60, 95, 98] (Fig. 1). Recent studies have specifically localized BDNF and TrkB to the CA3-CA1 axis in the hippocampus as necessary for the antidepressant effects and underlying ketamine induced synaptic potentiation [98]. Here, selective deletion of BDNF or TrkB in presynaptic CA3 or postsynaptic CA1 regions of the Schaffer collateral pathway, demonstrated BDNF-TrkB signaling in CA1 was required for the rapid synaptic and behavioral action of ketamine while revealing a specific synaptic locus for ketamine’s rapid antidepressant effects [98]. In separate work, the requirement for BDNF-TrkB has been proposed to be due to ketamine, as well as conventional antidepressants, to directly bind to the transmembrane region of TrkB and facilitate BDNF release. While this work suggests a common mechanism for all antidepressant action, it is not entirely clear how direct binding to TrkB would account for the differences in timing of the effects between rapid and conventional antidepressants [103].

## Mechanisms underlying long term maintenance of antidepressant effects

Recent studies have begun to uncover how intracellular signaling mechanisms that produce the acute antidepressant action of ketamine transition to a sustained effect that may last over a week [104]. This work elucidated several key aspects of sustained ketamine action. First, the acute translation-dependent effect of ketamine is required to trigger its sustained effect. Second, the sustained effect relies on transcriptional regulation, which is elicited by the initial synaptic plasticity. Finally, the transcriptional mechanisms underlying sustained ketamine effect requires the specific function of Methyl CpG binding protein 2 (MeCP2).

MeCP2 is a transcriptional regulator that controls gene expression both at the level of individual genes and via modulation of larger chromatin structure [105, 106]. MeCP2 dependent transcriptional processes also play critical roles in synaptic plasticity and neurotransmission [107–109]. MeCP2 can be functionally regulated by phosphorylation at several sites [110]. However, phosphorylation of MeCP2 at Serine 421 (Ser421) is neuronal specific regulated by activity such as postsynaptic calcium signaling and calcium-calmodulin kinases including phosphorylation of CaMKII at Threonine 286 (Thr286) [111]. Activity dependent processes have been shown to selectively induce MeCP2 Ser421 phosphorylation in the brain and thereby regulate MeCP2 dependent functions including spine maturation [106, 111–113].

As stated above, recent findings demonstrated a causal link between initial homeostatic neuroplasticity events elicited by ketamine and subsequent transcriptional mechanism that sustains ketamine’s behavioral effects. According to these results, the ketamine elicited AMPA receptor dependent synaptic potentiation induces a subsequent shift in excitation-inhibition balance towards excitation that ultimately leads to increases in MeCP2 Ser421 phosphorylation. Previous work has shown an increase in MeCP2 Ser421 phosphorylation broadly alters the MeCP2-dependent transcriptional landscape and thus acts as a global regulator of transcription, and likely impacts expression of target genes [106]. In agreement with this premise, in the context of ketamine action MeCP2-dependent transcription may augment long-term synaptic adaptations, in particular the magnitude of the synaptic potentiation seen in animal models and augmented ketamine responses seen in patients after repeated ketamine administration.

Acute subanesthetic ketamine increases MeCP2 Ser421 phosphorylation in the hippocampus a week after treatment but not in the initial hours after injection when the rapid antidepressant response occurs. These effects were specific to MeCP2 and were not paralleled by changes in other transcriptional factors [104]. The requirement for MeCP2 Ser421 phosphorylation in the sustained antidepressant action of ketamine was tested specifically in Mecp2 S421A knock-in mice, in which Ser421 was converted to an alanine such that endogenous MeCP2 is no longer phosphorylated at this site. MeCP2 S421A knock-in mice administered an acute injection of ketamine displayed rapid antidepressant-like effects, however, the sustained antidepressant action was no longer observed. The observation that MeCP2 Ser421 phosphorylation is required for prolonged but not rapid antidepressant action has a precedent in earlier work on classical antidepressants [114]. Based on current findings, one can propose a mechanistic model in which the initial translation dependent effects of ketamine [60, 95] are maintained via activation of a positive feedback loop where enhanced synaptic potentiation maintains enhanced transcriptional activity to prolong the initial effect. However, these findings and the associated models leave open the question why and how this positive feedback loop eventually gets broken and the antidepressant effect diminish with time.

As stated above, this initial and rapid antidepressant protein translation component involves the rapid increase in BDNF protein synthesis which drives the downstream intracellular signaling of MeCP2. Endogenous BDNF is also a potent activator of mTOR, which had previously been proposed to trigger rapid antidepressant action. Previous work demonstrated that ketamine’s effects on mTOR are not initial engagement at the synapse but rather downstream of BDNF and are not required for the rapid antidepressant effects [54]. This data agrees with recent clinical work showing that rapamycin, an mTOR inhibitor, does not attenuate ketamine’s rapid antidepressant action but may prolong it. It is possible that mTOR signaling is linked in some way to the intracellular signaling pathway that triggers downstream transcriptional MeCP2 processes to produce sustained processes. Further studies are needed to further dissect this intracellular signaling pathways and determine whether MeCP2 and mTOR are linked.

The involvement of MeCP2 is also particularly intriguing as it plays a critical role in spine maturation [106, 111–113]. A recent study found that spinogenesis is critical in sustaining the antidepressant action of ketamine but not in its rapid effects. Spinogenesis is well studied in early development, however these initial spines are not functional and undergo synapse maturation to become functional. The ketamine mediated structural effect on spinogenesis requires intracellular signaling, which may involve MeCP2 processes, in order to produce functional changes on neurotransmission that mediate the sustained action. Collectively, taken together these data strongly support further investigation into the intracellular signaling of MeCP2 and its links to mTOR and spinogenesis in order to elucidate potential points for therapeutic drug discovery. While preclinical studies provide a framework to investigate mechanisms of ketamine action, clinical studies have also provided mechanistic insight.

## Translational studies examining preclinical mechanisms

Clinical studies showed ketamine transiently increases cortical glutamate release in a manner that is related to the magnitude of its antidepressant effects. Ketamine increases nearly every index of cortical activity in healthy humans including glucose metabolism measured with ^18^F-deoxyglucose-PET [115, 116] and MR-based measures of oxygen metabolic rate and cerebral blood flow [117]. Early!H-MRS studies in healthy subjects described ketamine-related increases in voxel glutamate or glutamine levels in frontal cortex that were associated with the transient emergence of psychotic symptoms or cognitive impairment [118, 119]. Subsequently, a ^13^C-MRS involving the infusion of ^13^C-glucose as a metabolic tracer, in a mixed group of healthy subjects (*n* = 14) and MDD patients (*n* = 7), described a 13% increase in ^13^C-glutamine enrichment, i.e., an increase in V_Cycle_, that correlated with the emergence of dissociation symptoms [70]. This study was not powered to detect differences in glutamate release in the depressed patients and healthy subjects. Ketamine increases in glutamate release were linked to antidepressant response using PET mGluR5 imaging [120]. The paradigm builds on evidence that glutamate released by ketamine can reduce ligand (^11^C-ABP688, ^18^F-FPEB) binding to mGluR5 in two ways, through direct competition of glutamate and the PET ligand and by stimulating mGluR5 internalization [121]. Consistent with the ^13^C-MRS study, ketamine produced a 14% reduction in ^11^C-ABP688 binding in depressed patients and an 19% reduction in ligand binding in healthy subjects. In the depressed patients (*n* = 13), reductions in hippocampal ligand binding correlated (*r* = 0.52, *p* = 0.035) with improvement in depression [120].

The involvement of BDNF dependent homeostatic synaptic plasticity may account for the observation that extracellular glutamate levels surge after ketamine administration [70] and the increase in glutamate release correlates with the magnitude of the antidepressant response [120]. Several previous studies have shown homeostatic synaptic plasticities that elicit rapid dendritic BDNF synthesis are also coupled to augmentation of presynaptic glutamate release probability presumably via retrograde action of dendritically released BDNF [116]. In the hippocampus, there is also strong evidence that BDNF-TrkB signaling can regulate presynaptic release probability [122]. Therefore, it is plausible to expect that ketamine-induced increases in BDNF levels may augment presynaptic glutamate release in a manner that scales with the efficacy of BDNF signaling as postulated by clinical studies.

In animals, pretreatment with AMPAR antagonists prevents the emergence of the antidepressant effects of ketamine [123] and is tied to the induction of a specific form of homeostatic synaptic plasticity. While there is much discussion on plasticity as a mechanisms of ketamine action, there are different types of plasticities and specificity is important. For example, psychoplastogens is a term coined to highlight fast-acting therapeutics that produce rapid changes in synaptic plasticity. However, by this definition psychostimulants such as cocaine and amphetamine are psychoplastogens although one would not advocate for their use as rapid antidepressants for the treatment of depression. Instead, it is important to focus on the specifics of the type of plasticity which could potentially be harnessed by other drugs in advancing therapeutic options. While there is strong agreement on homeostatic synaptic plasticity underlying the mechanism of rapid antidepressant action, this hypothesis has yet to be tested directly in humans.

As noted earlier, the antidepressant effects of ketamine in animals is associated with activation of mTORC1 (increases in pmTOR/mTOR). When directly injected into the brain, the mTOR antagonist, rapamycin, attenuates the antidepressant effects of ketamine [87]. In contrast, intraperitoneal administration of rapamycin does not impact ketamine’s rapid antidepressant action suggesting mTORC1 was not required for the rapid behavioral effects [60]. Rapamycin may produce complex time-dependent effects on mTOR, including activation [124]. This might help to explain why pretreatment with a low dose of rapamycin (6 mg, po) two hours prior to ketamine administration extended the duration of the antidepressant effects of ketamine rather than blocked them [125]. The role of mTOR in antidepressant action is also supported by evidence of antidepressant activity of a direct mTOR activator [126, 127].

Recent clinical studies have also suggested that the antidepressant effects of ketamine may be associated with restoration of synaptic efficacy. The clearest preliminary signal with respect to the restoration of synaptic efficacy comes from a MEG study that showed that a single dose of ketamine failed to modulate the magnitude of sensory evoked potentials in healthy subjects or non-responding patients. However, depressed patients who responded to ketamine showed an increase in the amplitude of their sensory evoked response [128, 129]. Ketamine also has been shown to restore deficits in cortical resting-state functional connectivity, as assessed with fMRI [130, 131].

To date there has been a single pilot study directly addressing whether the antidepressant response to ketamine is associated with restoration of synapse density [132]. This study reported on three groups of subjects: healthy subjects (*n* = 7), depressed patients without synaptic deficits (*n* = 6), and depressed patients with synaptic deficits (*n* = 6). Both groups of depressed patients improved with ketamine. However, only the deficit group showed increases in ligand binding to the SV2A tracer suggestive of increases in synaptic density and SV2A binding increases that correlated with improvement in depression.

## Future directions

The discovery of ketamine’s rapid antidepressant effects in patients with depression and treatment resistant depression fostered a renaissance in clinical as well as preclinical neuropsychiatry. Ketamine’s rapid clinical efficacy indicated that symptoms of depression can be alleviated in patients with long history of resistance to conventional treatments within hours. The rapidity of ketamine action focused preclinical studies on fast synaptic signaling mechanisms and away from structural alterations associated with longer treatments such as synaptic rewiring and neurogenesis. These findings may suggest that ketamine’s rapid antidepressant action is due to its ability to induce homeostatic plasticity and not necessarily to ‘fix’ depression. In other words, the production of homeostatic plasticity may be a ‘masking’ effect to alleviate symptoms of depression rather than fixing the underlying causes of the disorder.

The requirement for homeostatic plasticity to produce the behavioral effects of ketamine also provides a potential novel therapeutic opportunity. Would the identication of compounds that directly target homeostatic plasticity represent a new avenue for treatment? In preclinical studies, this hypothesis was tested by proof of principle experiments. Retinoic acid receptor activaction produces rapid upscaling of homeostatic plasticity similar to ketamine but does not involve the NMDA receptor or its intracellular signaling pathway [133]. The retinoic acid signaling pathway is not required for ketamine mediated antidepressant action but direct activation of this pathway produced rapid antidepressant-like effects. Together, these findings suggest that compounds that elicit this form of homeostatic upscaling is sufficient for antidepressant action although this hypothesis requires clinical validation.

Another critical aspect of research is sustaining the antidepressant effects of ketamine. One approach may be the administration of ketamine to elicit the rapid antidepressant effects and then specific targeting of the downstream signaling pathway to extend the antidepressant effects without the need for repeated ketamine. This type of approach may prove advantangeous in avoiding repeated ketamine dosing that may be required for the treatment of depression in the long-term.

As this article illustrates, studies aimed at addressing ketamine’s mechanism of action built a direct bridge between basic synaptic signaling mechanisms and clinical practice. For decades, neuropsychiatric studies have centered around “slow” neurotransmission predominantly carried out by monoaminergic neurotransmitters [134]. Today, ketamine action and the role of fast glutamatergic neurotransmission in the pathogenesis and treatment of mood disorders present a new set of opportunities and challenges. In order to fully harness the emerging potential of fast neurotransmission in neurotherapies, it will be critical to uncover novel therapeutics to target fast neurotransmission that do not impair its fundamental function in sensory processing as well as learning and memory processes. For this purpose, it is essential to tap into parallel pathways of signaling carried out by distinct forms fast neurotransmission as well as uncover multiple signaling mechanisms that co-exist within single synapses at nanoscale. These efforts will also continue to inspire and direct future studies aimed at other effective treatments for neuropsychiatric disorders, especially those with fewer side effects, rapid onset and sustained efficacy.
